# Editorial for the Special Issue on Micro/Nano Structures and Systems: Analysis, Design, Manufacturing, and Reliability

**DOI:** 10.3390/mi14020253

**Published:** 2023-01-19

**Authors:** Stelios K. Georgantzinos

**Affiliations:** Laboratory for Advanced Materials, Structures and Digitalization, Department of Aerospace Science and Technology, National and Kapodistrian University of Athens, 34400 Psachna, Greece; sgeor@uoa.gr

**Keywords:** microstructures, nanostructures, mechanics, additive manufacturing, quality, design, analysis, finite element analysis

## Abstract

The advancement of fundamental sciences in recent decades has led to an increased focus on the prediction of phenomena occurring at the micro and nano scales. Micro- and nanostructures have a wide range of applications in various fields, such as aerospace and automobiles, and are widely used in nano- and micro-sized systems and devices, such as biosensors, nanoactuators, and nanoprobes. The design of these structures relies on a complete understanding of their physical and mechanical behaviors. Mechanics plays a crucial role at the micro- and nanoscales, from the generation of nanostructures to the properties of nanocomposite materials and the manufacturing and design of machines, structures, sensors, actuators, fluidics, and more. This Special Issue aims to bring together high-quality papers that advance the field of micro- and nanostructures and systems through the use of modern computational and analytical methods, in conjunction with experimental techniques, for their analysis, design, manufacture, maintenance, quality, and reliability.

Micro- and nanostructures and systems have become increasingly vital in various fields, such as aerospace, automobiles, and biomedical engineering. These structures and systems, which are typically on the scale of micro/nanometers, possess unique mechanical and physical properties that make them valuable for a broad range of applications, such as biosensors, nanoactuators, nanoprobes, and micro/nano-electromechanical systems. However, the design, manufacturing, and reliability of these structures and systems necessitate a comprehensive understanding of their behavior at the micro- and nanoscales. Therefore, the analysis of micro- and nanostructures and systems is an active field of research, with the objective of developing reliable methods for the prediction and control of phenomena at these scales.

The residual stress is defined as the presence of stress in a thin-film material layer without any externally applied forces. It can be compressive or tensile, vary over extremely large ranges of values, and even exhibit changes in the sign of the stress state. The residual stress is highly dependent on a number of factors, including processing conditions used during the deposition, type of material system (thin-films and substrate materials), and other processing steps performed after the thin-film layer has been deposited, particularly those involving exposure to elevated temperatures. The origins of residual stress can involve several complex and interrelated factors. Therefore, there is still no generally applicable theory to predict residual stresses in thin films. This lack of information can make device design more time consuming, expensive, and risky. Huff [1] reviewed the topic of residual stresses in deposited thin-film material layers and their effect on device behavior in micro- and nano-system development. The review discusses the origins of residual stress, its impact on device behavior, and methods to measure, control, and mitigate the impact of residual stress in micro- and nano-system device design and fabrication.

The fabrication of three-dimensional (3D) nanostructures has gained considerable attention in various fields, such as physics, chemistry, engineering sciences, and biology, due to their superior functionalities in comparison to planar nanostructures. However, the fabrication of 3D nanostructures remains a challenging task. To further advance their applications in commercial devices, there is a need for reliable fabrication methods, improved control, and enhanced ability to integrate multiple functions. Geng et al. [2] reviewed in depth a powerful method for realizing 3D nanostructures through atomic layer deposition-assisted 3D assembly using various sacrificial templates. The aim is to provide a comprehensive understanding of the use of atomic layer assembly (ALA) in a variety of sacrificial templates for 3D nanostructures and to highlight recent advancements. The ultimate goal is to unlock the full potential of this method for use in nanodevice applications.

Research and development of additive manufacturing (AM) technology has been ongoing for nearly three decades, and microscale AM has emerged as one of the most rapidly growing areas within the field. Significant progress has been made in the development and commercialization of new and innovative microscale AM processes, as well as in the application of these processes in a wide range of fields. However, there are still major challenges in terms of design, materials, processes, and ability to fabricate true three-dimensional structures and systems at a microscale. For example, microscale AM fabrication technologies are subjected to certain limitations and constraints due to their small scale, which may require the use of specialized design methodologies to overcome them. Rogkas et al. [3] reviewed the main processes, materials, and applications of current microscale AM technology. The aim is to identify future research needs for this technology and to discuss the need for the introduction of a design methodology. This study focuses on the design aspects, highlighting the advantages and limitations of AM at the microscale, as well as the selection of processes and materials.

Despite being a promising technology, fifth-generation (5G) wireless communication has yet to be fully implemented. To achieve the goals of 5G standards, such as higher data rates and ultrahigh-definition video streaming, the use of the millimeter wave (mmWave) band is crucial. However, the mmWave spectrum poses several challenges, including high connection losses, short wavelength, and restricted bandwidth, as well as path-loss challenges. To overcome these challenges, an antenna with wide bandwidth, high gain, narrow steerable beam, high isolation, low side-lobe levels, and multiband features is required. To achieve this, researchers have employed various strategies and techniques in the traditional antenna design process to improve performance in terms of bandwidth, gain, and efficiency, and to reduce mutual coupling between closely co-located antenna elements in MIMO and arrays. The latest state-of-the-art techniques, such as metamaterials, parasitic patches, hybrid feeding, EBG structure, and the impact of slots with different geometrical shapes in the radiator, are discussed by Ahmad et al. [4]. This study briefly reviews mutual coupling reduction techniques and focuses on the role of reconfigurability. Finally, it reviews the challenges in the field of antenna design and potential solutions to solve these challenges.

Deployable structures have the ability to significantly alter their geometric shapes by switching between different lattice configurations. One way to achieve this is by using compliant mechanisms as the lattice units, which can help prevent wear and friction among multi-part mechanisms. Liu and Hao [5] introduced two unique deployable structures that are based on a programmable compliant bistable lattice. Several novel parameters are introduced in the bistable mechanism to better control its behavior. By adjusting these parameters, the programmable bistable lattice can be optimized for specific goals, such as a larger deformation range or higher stability. The first structure is designed for 1D deployable movement and consists of multiple series-connected bistable lattices. The second structure, a cylindrical deployable mechanism, is designed based on the curved double tensural bistable lattice in order to explore the 3D bistable characteristics. This study mainly focuses on four types of bistable mechanisms, which are obtained by dividing the long segment of traditional compliant bistable mechanisms into two equal parts and setting a series of angle data to them. The results of the experiments and the FEA simulations confirm the feasibility of these compliant deployable structures.

Zhang et al. [6] presented a new refractive index sensor structure, which consists of a metal-insulator-metal (MIM) waveguide with two rectangular baffles and a U-shaped Ring Resonator (USRR). The sensor’s transmission characteristics were theoretically investigated using the finite element method. The simulation results indicate that Fano resonance is a sharp asymmetric resonance generated by the interaction between a discrete narrow-band mode and a successive wide-band mode. This study further explored the formation of broadband and narrowband, and identified key factors that affect the sensor’s performance. The best sensitivity of this refractive-index sensor is 2020 nm/RIU and the figure of merit (FOM) is 53.16. The proposed sensor is promising for use in nanophotonic sensing applications.

Cesmeci et al. [7] evaluated the performance of five different magnetorheological micropump designs, including two of their proposed designs and three from existing designs found in the literature. Comparisons were made using physics-based simulations, utilizing the fully coupled magneto-solid-fluid interaction simulations in the COMSOL Multiphysics software. To ensure a fair and meaningful comparison, all designs were given the same material and geometric properties, and the simulations were run for one complete pumping cycle. The results revealed that the proposed flap and duckbill valve models were able to pump 1.09 µL and 1.16 µL, respectively, in 1 s, which was a higher output than the other existing micropump models. Furthermore, when the magnetic flux density was at its maximum at 0.5 s, the flap and duckbill valve models could pump almost twice as much fluid as some of the existing valve models. The proposed models also had response times that were nearly five times faster than some of the existing models. Finally, the proposed micropump models demonstrated improved performance compared to existing designs, with higher net fluid volume output, low leakage during the contraction and expansion phase, and faster response times. These findings have significant implications for a wide range of applications, including insulin dosing systems for T1D patients, artificial organs, organ-on-chip applications, and micro-cooling systems.

Zhang et al. [8] investigated the changes in residual stress that occur during the heating and solidification of SiCp/Al composites. A one-way Fluid-Structure Interaction (FSI) model was developed to simulate the solidification process of molten material. By using process parameters, the model was able to predict the temperature distribution, liquid- and solid-state material transformation, and residual stress. Additionally, this study analyzed the crack that initiates from the thermal stress in the recast layer and proposed a mathematical model for the crack tip stress. The results revealed a wide range of residual stresses, ranging from 44 MPa to 404 MPa. The model was validated by comparing its results to the experimental data from three points on the surface layer.

Ye et al. [9] presented a study on the use of deep-narrow grooves (DNGs) of nickel-based alloy GH4169 in the aerospace industry. The electrochemical milling (EC-milling) process is a popular method for manufacturing special structures, such as DNGs, as it is highly efficient and does not result in residual stresses, burrs, or tool wear. However, the current EC-milling process has the disadvantage of poor removal of electrolytic by-products in the inter-electrode gap (IEG), which affects the machining accuracy and surface quality. To address this issue, a novel tube tool with a wedged end face was designed to create a pulsating flow field in the IEG, which enhances the removal of electrolytic by-products and improves the machining quality of DNGs. The flow field simulations and the experimental results showed that the tube tool with a wedged angle of 40 degrees was most suitable for the EC-milling process and was able to produce DNGs with a width of 1.49 mm ± 0.04 mm, a taper of 1.53° ± 0.46°, and a surface roughness of 1.04 μm, along with a milling rate of 0.42 mm/min. Additionally, increasing the spindle speed and the feed rate further improved the machining quality of DNGs. This study concludes that the use of the novel tube tool with a wedged end face can significantly improve the machining quality of DNGs in the aerospace industry.

To understand the impact of thermodynamic conditions on hydromechanical cavitation, a modified Singhal cavitation model was developed by Wu et al. [10] that considers thermodynamic effects. Using this model, numerical simulations were performed on a full flow field of an automotive electronic water pump at different temperatures (25 °C, 50 °C, and 70 °C). The results revealed that the trend of the simulation and experimental values was consistent across all flow rates and met the requirements for cavitation analysis. As the temperature increased, the low-pressure area inside the impeller of the electronic pump decreased, resulting in a decrease in NPSHr and an increase in cavitation resistance. Additionally, as cavitation developed, the maximum pressure pulsation amplitude in the impeller channel gradually increased, leading to increased vibration in the pump.

Lee et al. [11] demonstrated a flip-chip μ-bump bonding technology for a millimeter-wave wireless communication application that uses indium phosphide (InP) and silicon carbide (SiC) substrates. The proposed process includes a SiO_2_-based dielectric passivation process, a sputtering-based pad metallization process, an electroplating bump process for creating a flat-top μ-bump shape, a dicing process without peeling off the dielectric layer, and a SnAg-to-Au solder bonding process. This process enables the fabrication of 10 mm long InP-to-SiC coplanar waveguide lines with 10 daisy chains interconnected by one hundred μ-bumps. The performance of the CPW lines is uniform with an insertion loss deviation within ±10% along with an average insertion loss of 0.25 dB/mm, while achieving return losses of more than 15 dB at a frequency of 30 GHz. Additionally, a resonant tunneling diode device was fabricated and its DC and RF characteristics were investigated.

Liu and Hao [12] proposed a new type of linear guiding mechanism to address the common issues associated with traditional slide rail guides. The proposed mechanism utilizes compliant flexible members to transfer motion, force, and energy and is designed in a cylindrical shape, with a central platform that moves along its axis. The mechanism is comprised of several in-parallel curved compound double parallelogram mechanisms (CDPMs) connected by decoupling parallelogram mechanisms. Nonlinear finite element analysis (FEA) was used to analyze the stiffness of the mechanism, and it was found that the decoupling mechanisms can significantly improve stiffness in the undesired movement directions while maintaining original stiffness along the axis of motion. The proposed mechanism was also tested using a 3D printed prototype, and the results showed good agreement with the FEA analysis with a maximum error of 9.76%.

Bhoi et al. [13] evaluated the effects of different parameters on commonly used coating materials for micro-milling applications on high-speed steel substrates. Four different coating materials were chosen: titanium nitride (TiN), diamond-like carbon (DLC), aluminum titanium nitride (AlTiN), and titanium silicon nitride (TiSiN). A 3D finite element model was created in Abaqus to analyze the Hertzian normal stress when a 4 N normal load was applied using a rigid ball with a radius of 200 µm. The maximum normal stress found in the model was 12,109 MPa, with a deviation of 2.63% from the analytical results. The results indicated that the TiSiN coating with a thickness of 3 microns is the most optimal for micro-milling applications as it has the least plastic equivalent strain in the substrate.

Tseni et al. [14] presented a novel technique for optimizing inspection, warranty, and maintenance policies for micromachines that experience wear degradation. Their model takes into account the costs of quality control, warranty periods, and maintenance, and determines the optimal combination of these factors to improve product reliability and quality. The proposed optimization tool provides a comprehensive solution for determining the best approach to the maintenance and quality management of micromachines. To the best of the authors’ knowledge, this is the first time a warranty period has been incorporated into an optimization model for micromachines in the open literature, and it can bring significant benefits to their quality promotion strategy.

In recent years, micro-annular beams have become more widely used, expanding the possibilities for laser processing. However, the current methods for generating these beams have limitations, such as a spot of energy at the center of the beam. Sun et al. [15] presented a new method for generating an annular beam using a Fresnel zone plate with an annular structure, which was machined using a femtosecond laser. The results showed that by focusing the beam, an annular beam without a spot in the center can be obtained, and the radius and focal length of the annular beam can be easily adjusted. Additionally, by using two annular Fresnel zone plates that are concentrically connected, a concentric double-ring beam in the same focal plane can be obtained. The simulation and experimental results were consistent, demonstrating the potential of this method for applications requiring nontraditionally shaped laser beams.

In conclusion, the analysis and fabrication of micro- and nanostructures and systems is an active field of research, with a focus on developing reliable methods for the prediction and control of various phenomena at these scales. The residual stress, 3D nanostructures, and microscale additive manufacturing are some examples of the challenges that need to be addressed in order to advance the applications of micro- and nanostructures and systems in various fields, such as aerospace, automobiles, and biomedical engineering. The studies discussed in this editorial have provided valuable insights and perspectives on the current state of the field and have highlighted the need for further research in order to unlock the full potential of micro- and nanostructures and systems for commercial use.
